# Fate of Antioxidative Compounds within Bark during Storage: A Case of Norway Spruce Logs

**DOI:** 10.3390/molecules25184228

**Published:** 2020-09-15

**Authors:** Tuula Jyske, Hanna Brännström, Tytti Sarjala, Jarkko Hellström, Eelis Halmemies, Jan-Erik Raitanen, Janne Kaseva, Lucas Lagerquist, Patrik Eklund, Juha Nurmi

**Affiliations:** 1 Natural Resources Institute Finland (Luke), Tietotie 2, FI-02150 Espoo, Finland; jan-erik.raitanen@helsinki.fi; 2 Natural Resources Institute Finland (Luke), Teknologiakatu 7, FI-67100 Kokkola, Finland; hanna.brannstrom@luke.fi (H.B.); eelis.halmemies@luke.fi (E.H.); juha.nurmi@luke.fi (J.N.); 3 Natural Resources Institute Finland (Luke), Kaironiementie 15, FI-39700 Parkano, Finland; tytti.sarjala@luke.fi; 4 Natural Resources Institute Finland (Luke), Myllytie 1, FI-31600 Jokioinen, Finland; jarkko.hellstrom@luke.fi (J.H.); janne.kaseva@luke.fi (J.K.); 5 Department of Chemistry, University of Helsinki, PO Box 55, FI-00014 Helsinki, Finland; 6 Johan Gadolin Process Chemistry Centre, Åbo Akademi University, FI-20500 Turku, Finland; lucas.lagerquist@abo.fi (L.L.); patrik.c.eklund@abo.fi (P.E.)

**Keywords:** antioxidant, bark, condensed tannin, forestry side-stream, stilbene, timber

## Abstract

Softwood bark is an important by-product of forest industry. Currently, bark is under-utilized and mainly directed for energy production, although it can be extracted with hot water to obtain compounds for value-added use. In Norway spruce (*Picea abies* [L.] Karst.) bark, condensed tannins and stilbene glycosides are among the compounds that comprise majority of the antioxidative extractives. For developing feasible production chain for softwood bark extractives, knowledge on raw material quality is critical. This study examined the fate of spruce bark tannins and stilbenes during storage treatment with two seasonal replications (i.e., during winter and summer). In the experiment, mature logs were harvested and stored outside. During six-month-storage periods, samples were periodically collected for chemical analysis from both inner and outer bark layers. Additionally, bark extractives were analyzed for antioxidative activities by FRAP, ORAC, and H_2_O_2_ scavenging assays. According to the results, stilbenes rapidly degraded during storage, whereas tannins were more stable: only 5–7% of the original stilbene amount and ca. 30–50% of the original amount of condensed tannins were found after 24-week-storage. Summer conditions led to the faster modification of bark chemistry than winter conditions. Changes in antioxidative activity were less pronounced than those of analyzed chemical compounds, indicating that the derivatives of the compounds contribute to the antioxidative activity. The results of the assays showed that, on average, ca. 27% of the original antioxidative capacity remained 24 weeks after the onset of the storage treatment, while a large variation (2–95% of the original capacity remaining) was found between assays, seasons, and bark layers. Inner bark preserved its activities longer than outer bark, and intact bark attached to timber is expected to maintain its activities longer than a debarked one. Thus, to ensure prolonged quality, no debarking before storage is suggested: outer bark protects the inner bark, and debarking enhances the degradation.

## 1. Introduction

Bark of coniferous trees is an important organic side-stream of wood processing industries. Bark is produced when trees are debarked before sawmill timber production or in pulp mills. In Finland alone, forest industries’ roundwood consumption totaled 71.1 million cubic meters in 2019 [1]. The amount of bark is about 10% of the roundwood volume [2]. The forest industry in Finland thus produces ca. 7 million cubic meters of bark annually [1], which is mainly used to generate heat and energy. Regarding the need for a transition from fossil-based economy to bioeconomy, bark has an untapped potential as a sustainable source for biochemicals and -materials.

Bark of Norway spruce trees (*Picea abies* (L.) Karst.) contains high amounts of polyphenolic compounds, including condensed tannins (CTs), stilbenes (mainly stilbene glycosides and their aglycones), lignans, and flavonoids. These phytochemicals show multiple biological activities, such as antifungal and antimicrobial protection and antifeedant activity, all known to be vital for tree defense [3,4,5]. Due to their antioxidative and protective functions, these compounds are of commercial interest. They could be valorized as antioxidants, antimicrobials, or preservatives in food and cosmetics, techno-chemical products, or pharmaceuticals by applying environmentally friendly extraction and fractionation, such as pressurized hot water extraction or supercritical fluid extraction, followed by further purification and modification [6,7,8,9,10].

For example, CTs of Norway spruce bark have been tested for various purposes, such as carbon foams, water purification and barriers, and preservatives in edible snacks. Carbon foams could be produced from a crude tannin extract without purification [11]. Cationized tannins have been used in water purification [12,13,14]. In preservative use, tannins efficiently prevented fat oxidation and gave a unique taste to meat snacks [15]. Stilbene glycosides (*trans*-astringin, *trans*-isorhapontin, and *trans*-piceid) of Norway spruce bark, on the other hand, structurally resemble resveratrol that has gained an accumulating scientific evidence of its medicinal and other biological activities [16]. Bark-derived stilbenes with structural similarities to that of resveratrol are therefore of interest for refining purposes [17].

Evidence exists that the content and composition of bark metabolites varies within bark layers, between individual trees and forest stands, as well as among the geographical origins of tree populations [18,19,20]. These biological variations in bark quality may, however, be rapidly exceeded by the more pronounced property changes due to terminal and logistical operations, e.g., storage of biomass [21]. The current chains of operations from timber harvesting to logistics and wood processing at industrial sites are optimized for processes that utilize roundwood (i.e., sawing, pulping, energy uses). In contrast to wood, bark is chemically more complex [22] and prone to substance losses or changes in chemical composition during storage, i.e., after tree harvesting and post-debarking, the bark matrix is exposed to environmental and biological factors that may deteriorate the raw material [21,23]. During storage, extractives are lost through the hydrolysis by plant enzymes, by the action of wood colonizing organisms, and by oxidative processes. Volatile compounds, mainly monoterpenes, evaporate from woody biomass. The nature, magnitude, and the rate of changes in the content and composition of extractives depend on multiple factors including the types of harvesting, transportation, storage and the inventory-control systems of the wood used at the mill. Environmental conditions and properties of stored biomass, such as tree species, biomass assortment, and particle size all influence the changes. Bark compounds may degrade, polymerize, leach out, or even form toxic derivatives. Phenolic extractives are hydrophilic, and thus, in addition to microbial degradation reactions, they are also lost due to leaching [24,25]. Photodegradation reactions of phenolic compounds, such as stilbenes, may also occur during storage, as UV light and enzymatic activity catalyzes formation of phenoxy radicals, which being very unstable, lead to polymerization and cyclization reactions [17,26,27]. However, the changes in the composition and content of extractable phytochemicals within bark during storage remain poorly elucidated [28]. Furthermore, the concurrent changes in biological activities of bark extracts during storage are not fully understood. To create sustainable and economically feasible bark-based biorefineries, it is important to improve our understanding on the effects of long-term storage on the chemical quality of bark.

The aim of this study was to analyze the storage-time dependent changes in the content of major polyphenols of Norway spruce bark (i.e., CTs and stilbenes). We also analyzed the variation in antioxidative activity of the bark extracts during storage treatments. We tested the hypotheses that (1) changes in bark properties differ between the outer and inner layers of bark during the storage period (i.e., faster rate of changes in outer layer more prone to environmental factors); (2) seasonal variability exists in the rate of bark chemical modification during storage (i.e., higher rate during summer with warmer temperatures); and (3) bark stored on timber logs (i.e., before debarking) preserves its chemical content and antioxidative activity better/longer than the debarked raw material stored as industrial-scale bark piles. For the experiments, mature Norway spruce trees were harvested in the Ostrobothnia area in Finland, and the logs with bark were stored outside for a six-month monitoring period during winter and summer seasons. From individual sample trees, bark specimens were periodically collected for chemical and antioxidative activity analysis of the extracts. The study design is unique and to the best of our understanding, no similar long-term study has been conducted to monitor the fate of hydrophilic extractives of Norway spruce bark during storage.

## 2. Results and Discussion

### 2.1. Pre-Trial: Stilbene Glycosides of Bark at Sawmill during Storage

**Figure 1:**
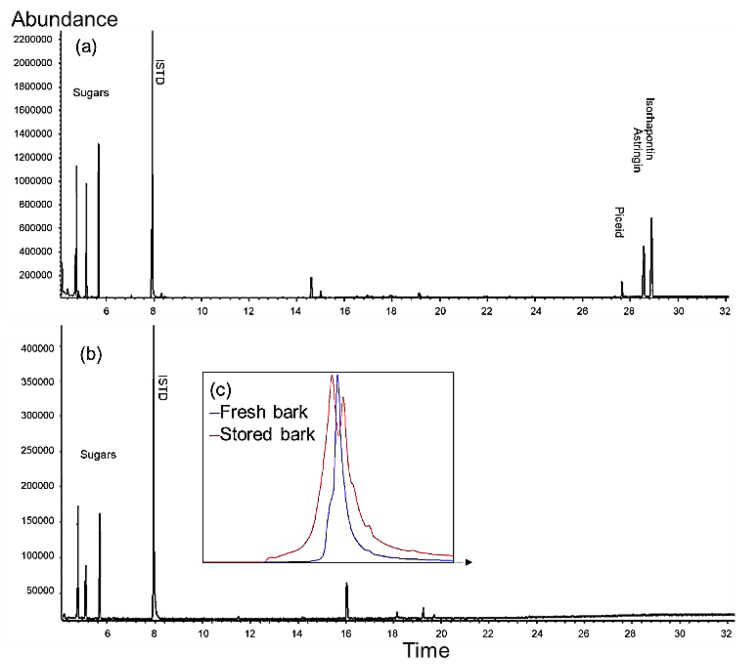
Gas chromatography–mass spectrometry (GC-MS) presenting the storage stability of stilbene glycosides of Norway spruce (*Picea abies* [L] Karst) bark: fresh bark at sawmill (**a**); 1-year-stored bark at a sawmill (**b**); and size exclusion chromatography results of fresh (blue line, unimodal) and 1-year stored bark (red line, bimodal) (**c**).

For a preliminary study of storage experiment, freshly debarked and older bark stored for ca. one year in the bark pile at sawmill were collected and their ethanol-water extracts qualitatively analyzed by gas chromatography-mass spectrometry (GC-MS) for the presence of stilbene glycosides (Figure 1a,b). According to the analysis, fresh bark contained all *trans*-stilbene glycosides typical of Norway spruce bark, namely piceid, astringin, and isorhapontin [9]. However, these compounds were not detected from the extracts obtained from the bark stored for a longer time period. Furthermore, analysis by size-exclusion chromatography indicated formation of both larger and smaller structures in bark that had been stored for ca. one year as compared to freshly collected bark from the sawmill (Figure 1c).

### 2.2. Yield of Stilbene Glycosides in Bark during Storage Experiment

**Figure 2:**
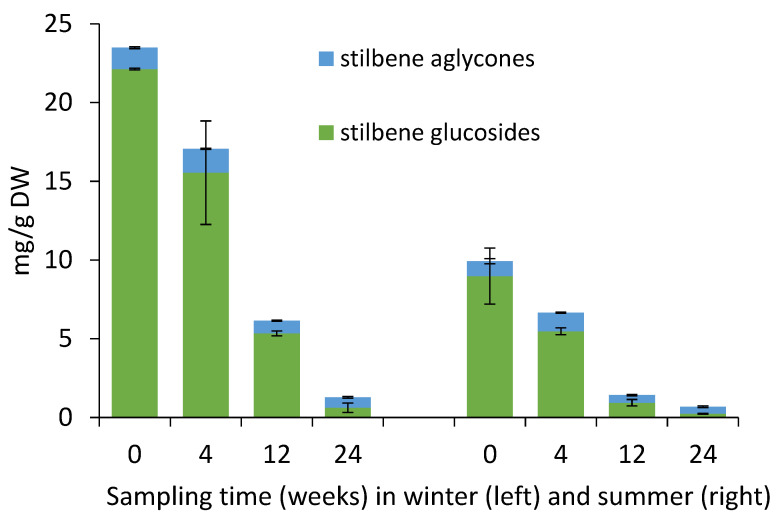
Yield of stilbene glycosides and aglycons in bark of Norway spruce saw logs during storage treatments in winter and summer 2017. The colors of bars indicate the stilbene compounds: green, sum of stilbene glycosides; blue, sum of stilbene aglycones. The whole bark was analyzed without separation into inner and outer layers.

According to our results from storage treatments, the total stilbene content in the bark of Norway spruce saw logs (i.e., the sum of stilbene glycosides and stilbene aglycones in whole bark) significantly declined during the storage (Figure 2, Table 1). It is known that environmental conditions have an effect on the microbial degradation of wood [23], as well as chemical reactions and physical processes (evaporation, leaching) leading to losses of extractives compounds [10,21]. Consequently, the rate of change in stilbene content decrease was higher in summer than in winter. In winter, since storage treatment onset, the total stilbene content was 73%, 26%, and 5% for weeks 4, 12, and 24 as compared to week 0 (Figure 2, Table 1). In summer, in contrast, the corresponding values were 77%, 14%, and 7%, respectively. Furthermore, the average stilbene glucoside amount was 2.8 times higher in winter samples than in summer samples (Figure 2, Table 1). For stilbene aglycons, the same trend was observed, with 1.5-fold amount in winter samples as compared to summer samples (Figure 2, Table 1). The reasons for the observed decline in the content of stilbene glycosides and aglycones in Norway spruce bark may include *trans* to *cis* - isomerization of the double bond and subsequent photocyclization reactions, as stilbenes are well-known for their reactivity and photosensitivity [9,17]. The stilbene compounds may also leach out, as they are water soluble and mostly located within the soft tissue layers of inner bark [18,19,20].

### 2.3. Yield of Condensed Tannins in Bark during Storage Experiment

**Figure 3:**
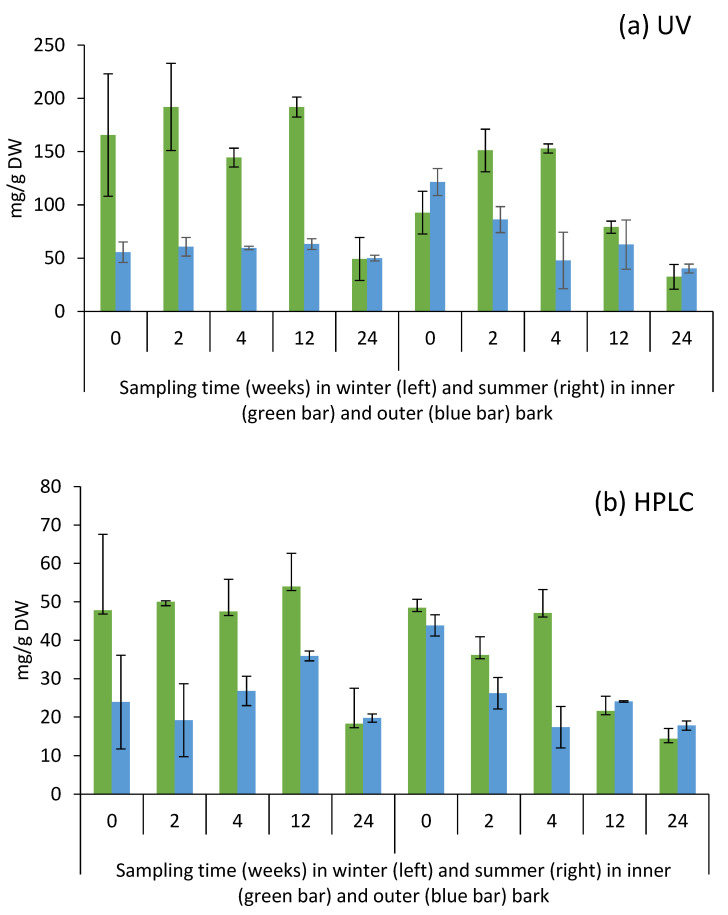
Yield of condensed tannins in inner and outer bark of Norway spruce saw logs during storage treatments in winter and summer 2017 as measured by the methods of UV spectrophotometry, λ = 280 nm (**a**) and high performance liquid chromatography (HPLC) after thiolytic degradation (**b**).

**Figure 4:**
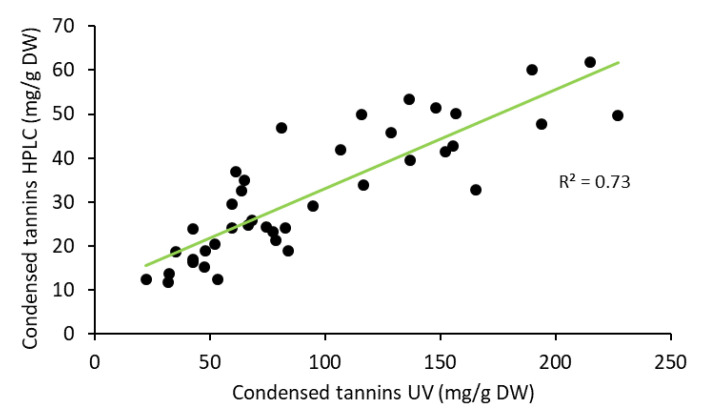
Correlation between the results of condensed tannin content analyzed by HPLC and UV methods.

The total condensed tannin content measured by UV-absorbance at 280 nm was clearly higher than the content determined by thioacidolysis (Figure 3) but a high correlation (*p* < 0.001) existed between the results from the two methods (Figure 4). This is understandable due to differences in the methods. The measurement of absorbance at 280 nm is commonly known as total phenolic index (TPI) since largely all plant phenolics have an absorption band at 280 nm and often with much stronger absorption intensity than tannins. However, both methods indicated quite a similar fate for tannins/phenolics during the storage. Generally, inner bark had higher tannin content than outer bark, which agrees with the results published by Krogell et al. [22] (Table 1). During winter, the tannin content remained rather steady for the first 12 weeks of storing but was considerably decreased after 24 weeks. During the last 12 weeks the decrease was especially notable for the tannin content of inner bark (Figure 3). During summer, the tannin contents appeared to decrease earlier than winter and outer bark tannins begun to fade immediately (Figure 3, Table 1). After 4 weeks, less than half of the original tannin content was determined in the outer bark after which the content remained quite stable. The tannin content of inner bark was quite stable for the first 4 weeks but decreased remarkably during the next 8 weeks and continued to decrease until the end of the storage (Figure 3).

It seems that the summer conditions accelerate the tannin degradation compared to winter conditions. Environmental factors in summer, i.e., higher temperature and higher relative humidity, may enhance chemical reactions involved in tannin degradation. Furthermore, metabolic activity of bark utilizing microbes is undoubtedly higher in summer than winter, and microbial degradation of tannins is likely to occur mostly in summer. The results also showed that during summer, tannins in outer bark degraded faster than in inner bark (Table 1). Obviously, outer bark gives a protective shield on inner bark which delays the degradation of tannins.

### 2.4. Composition of Condensed Tannins in Bark during Storage Experiment

Condensed tannins in spruce bark were mostly made of (epi)catechins but also (epi)gallocatechins were detected as minor subunits, especially in outer bark (Table 2). Already previous studies have shown that condensed tannins in Norway spruce are mostly procyanidins along with small proportions of prodelphinidins [15,29,30,31]. The average degree of polymerization was generally higher for tannins in inner bark than in outer bark (Table 2). Small proportions of condensed tannins with A-type linkages were detected in some samples. Proportion of (epi)gallocatechins (i.e., prodelphinidins) increased in the condensed tannins of outer bark during summer storing (Table 2). This may indicate that procyanidins in outer bark were more vulnerable to degradation than prodelphinidins. Other changes in the tannin profiles were not noticed during the storage period.

### 2.5. Molar Mass Distribution of Bark Extracts during Storage Experiment

**Figure 5:**
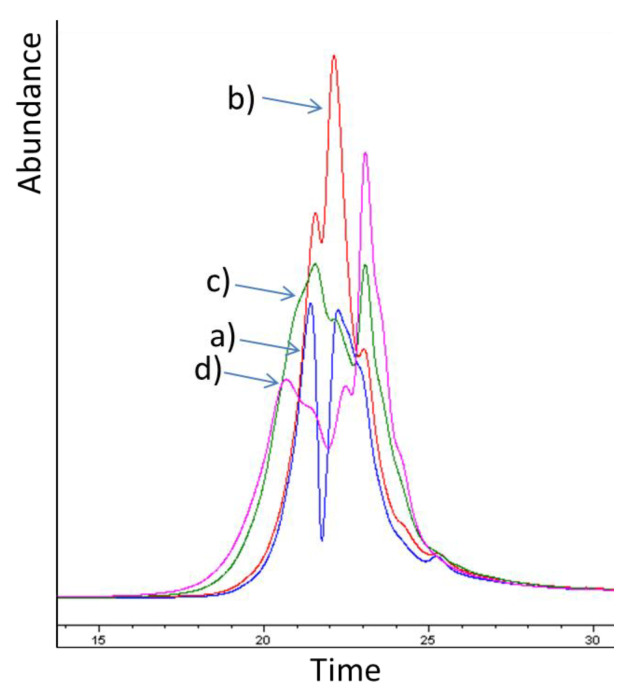
Example of size exclusion chromatogram of samples taken (**a**) 0-weeks (blue), (**b**) 4 weeks (red), (**c**) 12 weeks (green), or (**d**) 24 weeks (purple) after the onset of storage treatment.

**Figure 6:**
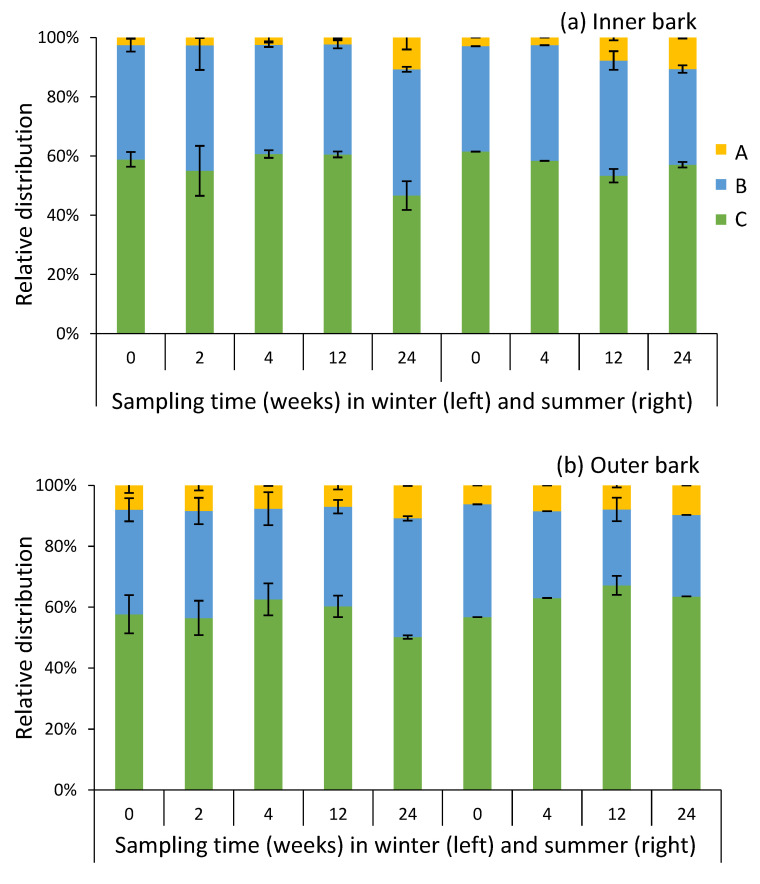
The relative distribution of compounds with different molar weights in 70% ethanol (70% EtOH) extracts of inner (**a**) and outer (**b**) bark of Norway spruce saw logs during storage treatments during winter and summer 2017 analyzed by HP-SEC. Regions A (yellow), B (blue), and C (green) represent the oligomeric and polymeric structures with molar masses of 15,000–1500 g/mol, 1500–500 g/mol, and 500–50 g/mol, respectively.

**Figure 7:**
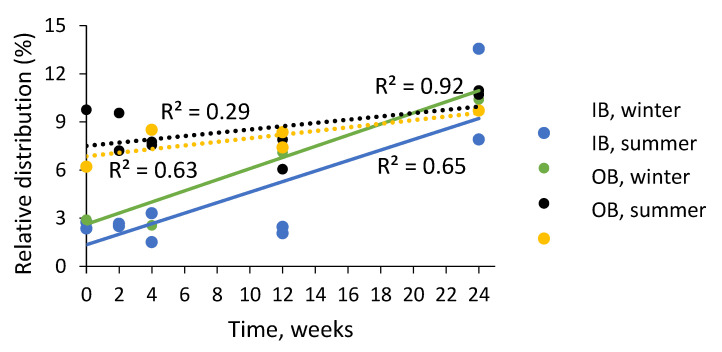
Relative distribution of oligomeric and polymeric compounds with molar weights of 15,000–1500 g/mol (i.e., region ‘A’ in Table 4 and Figure 6) in 70% ethanol (70% EtOH) extracts of Norway spruce saw logs during storage treatments during winter and summer 2017. IB, inner bark; OB, outer bark.

The ethanol extracts of inner and outer bark of saw logs were studied for their molar mass profiles during the storage experiments (Figure 5). The results showed that both in inner and outer bark, most of the compounds were of smaller molar mass (i.e., 1500–500 g/mol (region ‘B’) and 500–50 g/mol (region ‘C’) (Figure 6). Inner bark contained significantly less high molar weight compounds (i.e., 15,000–1500 g/mol, region ‘A’) than outer bark (Table 3). The storage of bark resulted in higher proportions of high molar weight compounds (region ‘A’), especially in the case of inner bark (Figure 5 and Figure 6, Table 3). For inner bark, the rate of change in compound ‘A’ proportion was also higher than that in outer bark during the experiment (Table 3). No significant difference in the average proportion of region ‘A’ compounds was found between winter and summer (Table 3). In summer, however, significantly higher proportion of compounds ‘A’ was found already on week 12 as compared to treatment onset (i.e., week 0), whereas in winter, a significant difference was found only after 24 weeks since the onset (Figure 7). Especially inner bark seemed to be prone to chemical reactions forming oligomeric and polymeric aromatic structures.

The reasons for changes in bark chemical quality during the storage and weathering include enzymatic reactions (e.g., living cell respiration) and biological degradation (due to various bacteria and fungi), as well as thermo-chemical oxidative reactions at the presence of adequate oxygen and moisture [23,24,26,32]. Day-light-induced processes may also have taken place, leading to the formation of polymers and smaller oligomers but also degradation of structures, especially in the case of photosensitive stilbenes [9,17,33,34].

### 2.6. Antioxidative Activity of Bark Extracts during Storage Experiment

**Figure 8:**
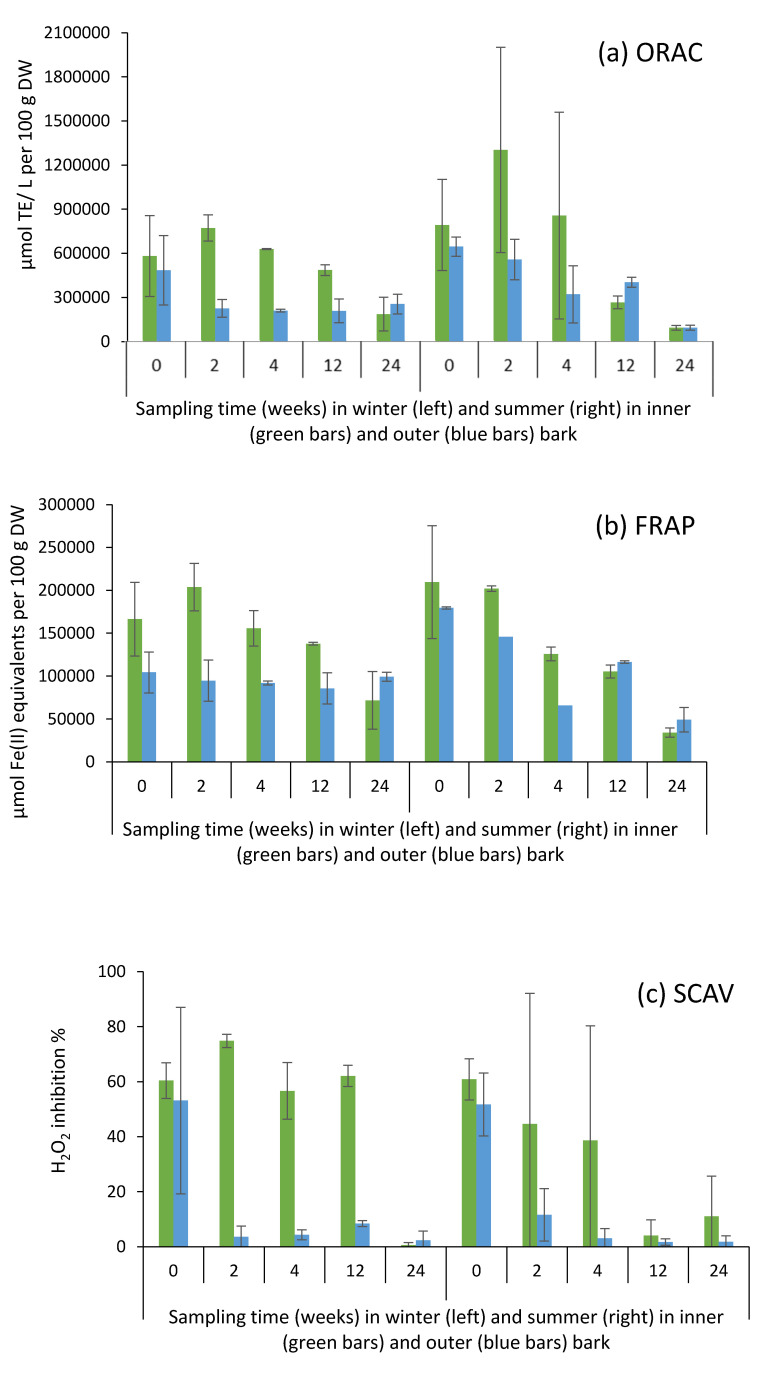
Oxygen radical absorbance capacity (ORAC; **a**), ferric ion reducing antioxidant power (FRAP; **b**), and hydrogen peroxide (H_2_O_2_) scavenging activity (SCAV; **c**) in inner and outer bark of Norway spruce saw logs after 70% ethanol (70% EtOH) extraction during storage treatments during winter and summer in 2017.

**Figure 9:**
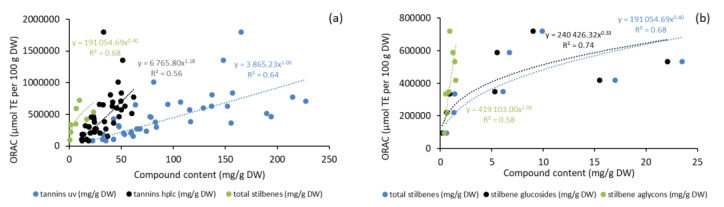
Oxygen radical absorbance capacity (ORAC) of Norway spruce bark (saw logs) as a function of total amounts of condensed tannins and stilbenes (**a**) and stilbene glycosides and aglycones (**b**) as analyzed by HPLC, UV and GC-MS methods during storage treatments in 2017.

The antioxidative capacity of extracts of Norway spruce bark (70% EtOH) obtained from saw logs (Figure 8 and Figure 9) and pulpwood (Figure S1) were analyzed by using three different techniques (i.e., ORAC, FRAP, and SCAV), which differ in their objectives [35]. All the studied bark extracts posed high antioxidant power. The results are consistent with previously published research indicating that spruce bark has much potential as a source of antioxidants amongst the studied wood species [9,36,37].

The storage of saw logs and pulpwood logs for several weeks during winter and summer resulted in significantly lower antioxidant capacity of bark extracts as observed with all the applied methods (Table 1 and Table 4; Figure 8 and Figure S1). In general, inner bark of saw logs exhibited significantly higher antioxidant capacity than outer bark (Table 1, Figure 8). The results also clearly indicate that inner bark of saw logs was able to maintain its antioxidative capacity for longer than outer bark did. Outer layers of bark may thus provide protection from the deteriorative factors (e.g., microbial, weather, mechanical) during the storage. For pulpwood, in contrast, no such a clear difference in antioxidative capacity was observed between the bark layers (Table 4, Figure S1).

No statistically significant difference was found in antioxidant activity between winter and summer; however, significant interactions for ‘season*storage time’ were found (Table 1 and Table 4). That is, during summer, the antioxidative capacity of bark extracts decreased with more accelerated rate as compared to winter conditions (Table 1 and Table 4; Figure 8).

As presented in Figure 9, antioxidative activity appeared to be well correlated with the content of phenolics (stilbenes and CTs) in bark extracts. The fairly high level of remaining antioxidative power of the bark extracts even after 12 to 24 weeks of storage treatment (especially in the case of inner bark in winter samples) indicates that despite hydrophilic bark extractives possibly leach out due to rainfall to some extent, a majority of the compounds still remained in the bark and may have been transformed into derivatives through oxidation, degradation, or polymerization, as indicated by our results (Figure 5 and Figure 6; Table 3). Our results thus provide evidence that the derivatives of bark hydrophilic extractives also have antioxidative properties.

Our study concentrated on the changes in bark chemical and antioxidative properties during the storage of un-barked saw logs and pulpwood (i.e., bark intact). In the parallel study of this, ca. 40% more extractives were found in the whole bark intact on saw logs than in the bark that had been removed from the logs and then stored separately in a bark pile for 24 weeks [38]. Furthermore, the content of stilbene glycosides (piceid, astringin, and isorhapontin) in bark pile decreased from ca. 17 mg/g DW to undetectable levels in only four weeks, while the stilbenes were still observed from the intact bark of saw logs [38]. More research is still needed on bark properties after the debarking process and during the storage of feedstock at industrial sites. It has been shown that conifer bark (*Picea abies*, *Pinus sylvestris,* and *Pinus radiata*) collected from pulp mills gave lower yields of CTs than the bark collected at sawmills [39]. In addition, the preprocessing of bark—milling and drying—can modify bark quality, e.g., fractionation according to bark particle size also led to fractionation of chemical composition [39]. In addition, the requirements for feedstock quality depend on the selected processing chain and end-use purposes of bark [40].

## 3. Materials and Methods

### 3.1. Bark Material

#### 3.1.1. Sample Trees and Experimental Design for Storage Treatments

**Figure 10:**
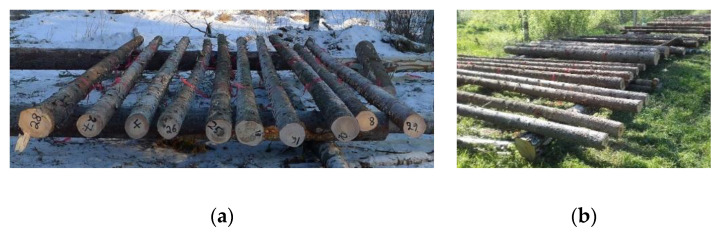
Single-stem set-up of storage experiment in winter (**a**) and summer (**b**) in 2017.

**Figure 11:**
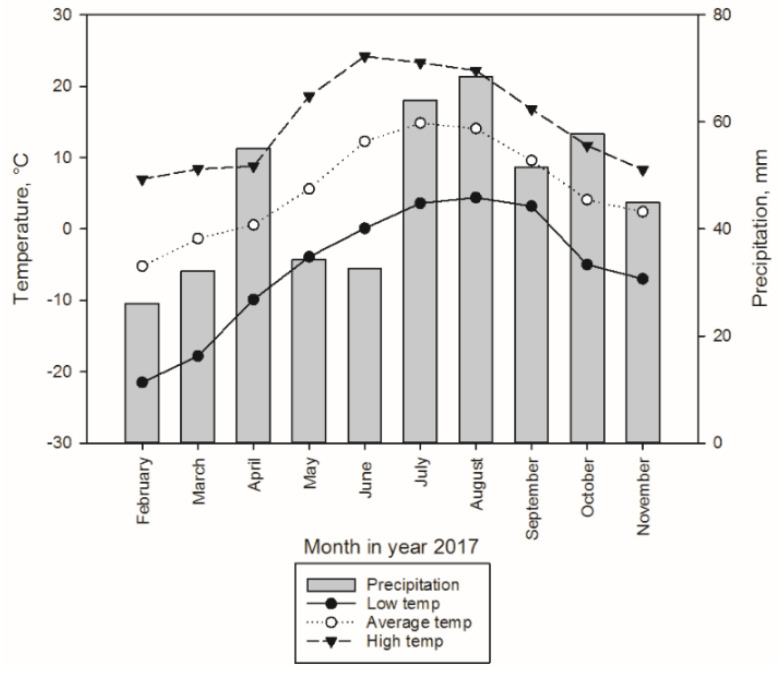
Monthly mean, minimum and maximum air temperatures (i.e., low, average, high, respectively) and monthly precipitation sum at the site during the experiment in winter and summer 2017.

Sample trees (altogether 40 trees) were harvested in 2017 from western Finland, Ostrobothnia region near Kokkola (63°54′44.0″ N, 23°25′17.0″ E). Norway spruce (*Picea abies* (L.) Karst.) trees were felled for the winter storage experiments on 6 February and the construction of experimental setup for storage study was finalized on 7 February. Similarly, for the summer storage study Norway spruce trees were felled on 29 May and the experimental setup was constructed on 30 May. All the trees for winter storage were cut by a harvester (incl. delimbing and cross-cutting). Saw logs for summer storage were similarly cut and delimbed by a harvester, whereas pulpwood trees were cut by a harvester and delimbed manually. The trees in saw log experiments were 56–119 years old, with an average diameter of 32 cm at 1.3 m stem height and a height of 206–270 dm (Table 5). The trees in pulpwood experiments were 38–103 years old, with an average diameter of 14 cm at 1.3 m stem height and a height of 100–153 dm (Table 6). The tree ages were calculated from the sample discs, which were taken from the remaining tree stumps. The saw logs and pulpwood stems were bucked to approximately 4.5 m and 5.0 m, respectively. The logs were numbered randomly and placed on a frame build of tree trunks so that their contact to the ground as well as their being buried under snow or vegetation during winter and summer, respectively, was prevented (Figure 10). The meteorological data (i.e., air temperature and precipitation) of the site during the experiment are presented in Figure 11. The data were obtained from the service by the Finnish Meteorological Institute [41].

#### 3.1.2. Industrial Bark

Norway spruce bark was provided by the sawmill (Timo Timber Oy) at Utajärvi (Northern Ostrobothnia region, Finland) in February 2017 (64°45′ N 026°25′ E). Both fresh bark and bark stored ca. one year in an industrial bark pile outside were collected. The fresh bark was obtained immediately after debarking. The one-year stored debarked bark was collected from underneath the uppermost layers of in the industrial bark pile. All the sampled material was kept at −20 °C prior to further analysis.

### 3.2. Sampling Design in Storage Treatments

**Figure 12:**
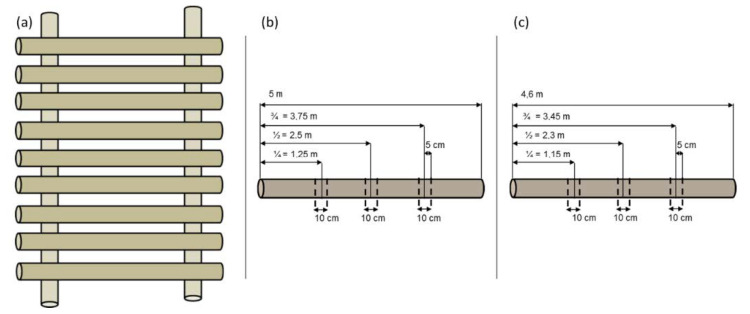
Experimental setup of single stem logs in storage experiment (**a**), and schematic presentation of the bark sampling from individual saw logs (**b**) and pulpwoods (**c**) for chemical and antioxidative analysis.

Bark samples were repeatedly collected from sampling locations (with no faults and defects) along the stem logs, as shown in Figure 12. The 10-cm wide sample discs were sawn with a chainsaw without chain oil. At each sampling time, three sample discs per tree were taken from four individual trees (i.e., two saw logs, two pulpwood logs) (Table 5 and Table 6). The samples were directly placed into the freezer as soon as possible and stored at −20 °C.

### 3.3. Preparation of Bark Extracts

For the chemical analysis of bark stilbenes and tannins, bark from frozen discs were separated and then first freeze-dried, after which bark was milled with a grinder (Moulinex, Groupe SEB, Ecully Cedex, France) into a smaller particle size of 0.5–1 mm for the analyses. Prior to the analysis, bark powder was stored at −80 °C. Industrial bark was milled and freeze-dried before analysis using a similar procedure. The extractions were carried out by using an accelerated solvent extraction (ASE) apparatus (Thermo Scientific Dionex ASE 350, Sunnyvale, CA, USA). The bark powder was extracted with hot water (75 °C, 100 MPa, 1 × 30 min static cycle) to yield extracts rich in CTs and stilbenes for their quantitative analysis. The bark powder was also separately extracted with EtOH/H_2_O (95:5, *v*/*v*) (EtOH, Altia, Rajamäki, Finland) to yield hydrophilic extracts (75 °C, 100 MPa, 1 × 30 min static cycle) for the analysis of the molar mass distribution.

For the quantitative analysis of bark stilbenes by GC-FID, bark from frozen discs were separated and freeze-dried, after which the bark was milled with a Retsch SM 100 cutting laboratory mill (Retsch GmbH, Haan, Germany) equipped with a bottom sieve with trapezoidal holes (perforation size <1.0 mm). Prior to the analysis, bark powder was stored at –20 °C. The extractions were carried out by using a Dionex ASE 100 instrument. The bark powder was extracted with hot water (120 °C, 100 MPa, 1 × 10 min static cycle) yielding extracts rich in hydrophilic CTs and stilbenoids for their quantitative analysis.

### 3.4. Chemical Analysis of Extracts

#### 3.4.1. Yield of Condensed Tannins

The phenolic concentration was expressed as milligrams of purified quebracho tannin equivalents to milligrams of dry extracts. It was assumed that the phenolic compounds in conifer bark extracts measured by spectrophotometric measurement primarily correspond to tannins, as reported by Bianchi et al. [30] and the expression “tannin yield” was used for the total phenolic compounds. Highly purified quebracho tannins (FINTAN QP, Silvateam S.p.A., San Michele Mondovì, Italy) was dissolved in 0.1 M NaOH. A series of different tannin concentration was prepared, and a calibration curve was plotted against UV absorbance measured at 280 nm using a UV-Vis spectrophotometer (Shimadzu UV-2600, Kyoto, Japan). The extract powder was dissolved in 0.1 M NaOH and the absorbance at 280 nm was measured and the tannin content was calculated.

#### 3.4.2. Chemical Composition of Condensed Tannins

Condensed tannin, i.e., proanthocyanidins, was determined by HPLC after thiolytic degradation according to Mattila et al. [42]. Briefly, freeze-dried samples were weighed (20–30 mg) into 1.5 mL Eppendorf vials and 1 mL of depolymerization reagent (3 g cysteamine/4 mL 13 M HCl/56 mL methanol) was added. The vials were sealed and incubated for 60 min at 65 °C, after which the degradation products, i.e., free flavan-3-ols (terminal units) and their cysteaminyl derivatives (extension units), were separated on Zorbax Eclipse Plus C18 column (Agilent Technologies, Inc.; Espoo, Finland, 2.1 × 50 mm, 1.8 μm) and determined by HPLC (Agilent 1290 Infinity, Agilent Technologies, Inc., Espoo, Finland) equipped with diode array detection (DAD) and fluorescence detection (FLD).

#### 3.4.3. Yield of Stilbene Glycosides

After extraction, approximately 3 mg of extract was placed in a test tube and evaporated to dryness under N_2_-stream. After drying, 0.5 mL of pyridine and 0.3 mL of the silylation reagent *N*-trimethylsilyl imidazole (TMSI in pyridine, Sigma-Aldrich, Merck KGaA, Darmstadt, Germany) was added and the sample was kept in a block heater at 70 °C for 60 min. The silylated extracts were analyzed qualitatively by GC–MS (Hewlett-Packard 5973 MSD, EIMS 70 eV; Agilent, Santa Clara, CA, USA) equipped with a Zebron ZB-5MSi capillary GC column (30 m × 0.25 mm × 0.25 µm). Identification of the peaks was done by comparing the fragmentation patterns with a commercial (NIST14/Wiley11) library, as well as the MS libraries available at our laboratory. Quantification of the identified stilbene glycosides (astringin, isorhapontin, piceid), and stilbene aglucones (sum of resveratrol, isorhapontigenin, and piceatannol) was done by GC-FID (Agilent Hewlett-Packard 6850) equipped with an Agilent HP-5 19091J-413 column (30 m × 0.32 mm × 0.25 μm) on silylated samples, using heneicosanoic acid (0.1 mg/mL; Sigma-Aldrich Chemie GmbH, Steinheim, Germany) and betulinol (0.1 mg/mL; Sigma-Aldrich, St. Louis, MO, USA) as internal standards. The samples were injected at 290 °C and were detected at 300 °C. The temperature program was at 100 °C (1.5 min), 6 °C/min to 180 °C, 4 °C/min to 290 °C (13 min), 4 °C/min to 300 °C (20 min).

#### 3.4.4. Analysis of Molar Mass Distribution of Extracts

The molar weight profile of the ethanol extracts was determined by High Pressure Size-Exclusion Chromatography (HP-SEC) using an Agilent 1100 Series HPLC instrument (Agilent Technologies, Waldbronn, Germany) equipped with a G1315B DAD-detector, 2 × Jordi Gel DVB 500A (300 mm × 7.8 mm) columns (Columnex LLC, New York, NY, USA; 40 °C), and a 50 mm × 7.8 mm guard column. This setup allows selective analyses of aromatic (polyphenolic) compounds. One percent AcOH (J.T. Baker, Deventer, Holland) in THF (Riedel-de Haën-Honeywell, Seelze, Germany) served as eluent at a flow rate of 0.8 mL/min with 35 min analysis time/sample. The samples stored in a freezer were first allowed to thaw 2 h before sample preparation. The samples (3 mg) were dissolved in the eluent solution (1.5 mL) to yield a concentration of 2 mg/mL. The samples were then vortexed for 0.5–1 min and filtered with a 45 µm PTFE-filter to remove any insoluble particles prior to analysis. It should be noted that only the soluble part of the extract is analyzed, and the results cannot represent the molar weight profile of the whole physical sample as all sample contained minor insoluble particles.

The relative distribution of compounds with different molar weights was calculated from the integrals of the HP-SEC chromatograms. The whole signal from ranging from 15–27 min were split into region A (15–20 min) representing oligomeric and polymeric structures with molar masses 15,000–1500 g/mol, region B (20–22 min) representing molar weights 1500–500 g/mol and region C (22–27 min) representing molar weights 500–50 g/mol. For the calibration of molar weight, a commercial polystyrene standard was used.

### 3.5. Antioxidative Analysis of Extracts

Antioxidant properties of the extracts were assessed by indirect methods to cover different antioxidant mechanisms. Indirect methods included a single electron transfer-based (SET) and a hydrogen atom transfer-based (HAT) (FRAP and ORAC) and radical scavenging assays (SCAV).

#### 3.5.1. FRAP

Differences in the antioxidant activity between inner and outer bark extracts obtained from the storage experiments were measured using a SET-based FRAP (Ferric ion reducing antioxidant power) method, which measures the ability of an antioxidant to reduce ferric (FeIII) to ferrous (FeII) ions [43]. The reaction mixture contained the sample, 20 mM FeCl_3_·6H_2_O (Sigma-Aldrich Chemie GmbH, Steinheim, Germany) and 10 mM 2,4,6-Tris(2-pyridyl)-s-triazine (TPTZ) (Sigma-Aldrich Chemie GmbH, Steinheim, Germany) in 300 mM acetate buffer pH 3.6. The formation of ferrous-tripyridyltriazine complex in the reaction mixture is measured by absorbance at 593 nm in 96-microplate format with three technical replicates of each sample on the plate and series of dilutions to fit the sample to the standard curve. FeSO_4_·7H_2_O (Sigma-Aldrich Chemie GmbH, Steinheim, Germany) was used as a standard compound and l(+)-ascorbic acid (150 μM and 800 μM) (VWR Chemicals) as a control and the results are expressed as μmol/L Fe(II) equivalents.

#### 3.5.2. ORAC

The Oxygen Radical Absorbance Capacity (ORAC) assay is a HAT based method, which measures the oxidative dissociation of fluorescein at the presence of peroxyl radicals (R-O-O%), which causes reduction in the fluorescence signal. The antioxidant’s protective ability is based on the inhibition of the breakdown of fluorescein caused by the peroxyl radicals. The assay was modified from the method described by Huang et al. [44] and Prior et al. [45] and carried out in 96-well format with two technical replicates of each sample on the plate. Each reaction mixture contained 25 μL of the sample in 0.075 M phosphate buffer pH 7.5 (Merck), 150 μL of 8.16 × 10^−5^ mM fluorescein (Sigma-Aldrich Chemie GmbH, Steinheim, Germany) and 25 μL of 2,2′-Azobis(2-methylpropionamidine) dihydrochloride (Sigma-Aldrich Chemie GmbH, Steinheim, Germany). For each sample, a protocol with a series of five dilutions (1:1–1:320) was used and additional dilutions if needed to adjust the sample concentration to the standard curve. 0.153 mM Trolox ((±)-6-Hydroxy-2,5,7,8-tetramethylchromane-2-carboxylic acid, vitamin E analog) (Sigma-Aldrich Chemie GmbH, Steinheim, Germany) was used as a standard compound and the results are expressed as Trolox equivalents (μmol/L TE). Vitamin C (l(+)-ascorbic acid; Merck KGaA, Darmstadt, Germany) was used as a reference compound.

#### 3.5.3. SCAV/FOX Reagent Method

The hydrogen peroxide (H_2_O_2_) scavenging activity, based on transition metal chelation, was determined by using a method modified from Hazra et al. [46] and Jiang [47] with microplate reader in 96-well format with four technical replicates on each plate. An aliquot of 2 mM H_2_O_2_ (Merck KGaA, Darmstadt, Germany) was added to the reaction mixture with the sample, 2.56 mM ammonium iron (II) sulphate·6H_2_O (BDH Prolabo) and 111 μM xylenol orange disodium salt (Sigma-Aldrich Chemie GmbH, Steinheim, Germany). After 30 min incubation, the absorbance of ferric-xylenol orange complex at 560 nm was measured. The assay measures the ability of the sample to scavenge H_2_O_2_ and prevent the oxidation of Fe(II) to Fe(III) which is indicated by the formation of ferric-xylenol orange complex. The H_2_O_2_ scavenging ability is expressed as inhibition percentage (%) of Fe(II) oxidation to Fe(III). Sodium pyruvate (Sigma-Aldrich Chemie GmbH, Steinheim, Germany) was used as a reference compound.

### 3.6. Statistical Analysis

To analyze the statistical differences in chemical constituents and antioxidative activities of the bark extracts between storage times (i.e., storage time in weeks since the onset of the experiment), seasons (winter vs. summer), and bark materials (inner vs. outer bark), a hierarchical linear mixed models were fitted to the data with the assumption of the normal distribution for CTs, distributions of molar mass of the compounds (i.e., regions ‘A’, ‘B’, and ‘C’). Logarithmic transformation (ln) was used for skewed dependent variables, and also to normalize variation within time points (stilbenes, ORAC, FRAP, and SCAV assay data). The analysis of stilbenes was simplified because of the small data set having only both main effects in the model. Tree was used as a random variable to account the variation within two bark materials of a single tree avoiding pseudo-replication. The method of Bonferroni was used in multiple comparisons with a significance level of α = 0.05. The residuals were checked for normality using graphical figures. The models were fitted using the MIXED procedure of IBM SPSS STATISTICS (v. 25) with a restricted maximum likelihood estimation method (REML).

## 4. Conclusions

To conclude, our results clearly demonstrate that the analyzed hydrophilic extractives of Norway spruce bark (i.e., condensed tannins and stilbene glycosides and aglycons) rapidly declined in content during the storage of timber logs. This was the case for both larger dimensional saw logs and smaller diameter pulpwood. Outer bark appeared to protect the inner bark, thus decelerating degradation, modification, and possible leaching of the valuable compounds during storage. Therefore, debarking would lead to more rapid degradation processes of the valuable compounds. Based on the results, we conclude that for optimizing bark quality prior to its processing in bark-based biorefineries, debarking should be postponed, and bark stored intact on logs instead of debarked feedstock in piles. In order to prolong the quality of bark feedstock for recovery of value-added compounds via, e.g., extraction processes, no debarking before the storage is therefore suggested. Our study showed that stilbenes rapidly degraded during storage, whereas tannins were more stable. Only ca. 5–7% of the original stilbene amount was detected after 24-week storage (whole bark analyzed). On average, ca. 44% of the original amount of condensed tannins (analyzed by HPLC) were found after the 24 weeks of storage (ca. 34% and 62% of the original amount in inner and outer bark, respectively). The weather conditions during summer with higher atmospheric temperature and higher precipitation seem to accelerate the deterioration processes of bark chemical properties during storage as compared to winter with lower temperature and moisture level. For example, on average 35% and 61% of the original amount of condensed tannins (by HPLC) remained in the bark in summer and winter, respectively, after 24-week-storage period. Changes in antioxidative activity of the bark extracts were analyzed by three different assays in order to cover the different antioxidant mechanisms more widely than by relying on a single method only. The results of the assays showed that on average, ca. 27% of the original antioxidative capacity remained 24 weeks after the onset of storage treatment, while a large variation (ca. 2–95% of the original capacity remaining) in results between the different assays, seasons and bark materials (i.e., inner vs. outer layers) was detected. In summer, the remaining antioxidant power after 24-week storage was on average 15% of the original one, while that for the winter season was even as high as 38%. Furthermore, it seems that the changes in antioxidant activities were, at least partly, less pronounced than the changes obtained for analyzed chemical compounds, indicating that the derivatives of the compounds also contribute to the antioxidant capacity of bark.

## Figures and Tables

**Table 1:** Results (*p*-values) from testing the statistical differences between storage time (i.e., weeks after the treatment onset), season (i.e., winter and summer 2017), and the layers within bark (i.e., inner and outer bark) in terms of the quantitative amounts of stilbene glycosides and aglycones, condensed tannins analyzed with HPLC and spectrophotometric methods (CTs HPLC, CTs UV); and the analysis of antioxidative activities of bark extracts (70% EtOH) of Norway spruce saw logs analyzed with ORAC, FRAP, and SCAV tests. Interaction terms are indicated by x.

	*p*-Values, Saw Logs						
Factor	Stilbene Glycosides	Stilbene Aglycones	CTs HPLC	CTs UV	ORAC	FRAP	SCAV
Storage time	0.003	0.007	0.005	0.000	0.001	0.000	0.031
Bark layer	n.d.	n.d.	0.000	0.000	0.001	0.001	0.025
Season	0.009	0.010	0.140	0.052	0.526	0.386	0.275
Storage time × Bark layer	n.d.	n.d.	0.001	0.003	0.145	0.002	0.465
Storage time × Season	n.d.	n.d.	0.061	0.152	0.089	0.006	0.737
Bark layer × Season	n.d.	n.d.	0.003	0.001	0.177	0.287	0.543
Storage time × Bark layer × Season	n.d.	n.d.	0.021	0.030	0.730	0.399	0.563

**Table 2:** Composition and properties of proanthocyanidins in Norway spruce bark (saw logs) samples during the storage treatments in winter and summer 2017.

Season	Time Weeks	Sample	DP ^1^	PC ^2^ (%)	PD ^3^ (%)	A-type ^4^ (%)
W	0	IB	8.2 ± 0.7	100.0	n.d.	n.d.
W	2	IB	8.7 ± 0.8	100.0	n.d.	n.d.
W	4	IB	7.4 ± 1.0	99.9	0.2	n.d.
W	12	IB	8.0 ± 1.8	100.0	n.d.	n.d.
W	24	IB	8.3 ± 2.8	95.5	4.5	n.d.
W	0	OB	6.1 ± 0.4	83.4	16.6	1.5
W	2	OB	6.4 ± 0.4	90.5	9.5	n.d.
W	4	OB	6.2 ± 0.8	83.8	16.2	n.d.
W	12	OB	6.9 ± 1.8	84.8	14.2	n.d.
W	24	OB	6.6 ± 0.4	86.0	14.1	1.3
S	0	IB	7.1 ± 0.1	100.0	n.d.	2.8
S	2	IB	5.6 ± 0.6	99.2	1.6	5.6
S	4	IB	6.2 ± 0.7	100.0	n.d.	3.6
S	12	IB	8.0 ± 0.4	97.1	2.9	2.2
S	24	IB	6.2 ± 0.3	95.2	4.8	n.d.
S	0	OB	5.9 ± 0.4	93.6	6.5	2.6
S	2	OB	5.5 ± 0.6	91.2	8.8	2.7
S	4	OB	7.0 ± 0.2	80.4	19.7	1.6
S	12	OB	7.5 ± 0.5	84.1	16.0	n.d.
S	24	OB	6.7 ± 0.1	81.7	18.3	n.d.

^1^ DP = mean degree of polymerization; ^2^ PC (%) = procyanidins, i.e., (epi)catechin units proportion; ^3^ PD (%) = prodelphinidins, i.e., (epi)gallocatechin units proportion; ^4^ A-type (%) = A-type bonding proportion from all bonding types (A-type + B-type); W, winter season; S, summer season; IB, inner bark; OB, outer bark; n.d., not detected.

**Table 3:** Results (*p*-values) from testing the statistical differences between storage time (i.e., weeks after the treatment onset), season (i.e., winter and summer 2017), and the layers within bark (i.e., inner and outer bark) in terms of relative distribution of compounds with different molar weights in 70% ethanol (70% EtOH) extracts of Norway spruce saw logs during storage treatments in winter and summer 2017 analyzed with HP-SEC. Regions A, B, and C represent the oligomeric and polymeric structures with molar masses of 15,000–1500 g/mol, 1500–500 g/mol, and 500–50 g/mol, respectively. Interaction terms are indicated by x.

	*p*-Values, Saw Logs		
Factor	A	B	C
Storage time	0.002	0.568	0.143
Bark layer	0.001	0.002	0.138
Season	0.337	0.141	0.257
Storage time × Bark layer	0.059	0.192	0.382
Storage time × Season	0.171	0.279	0.248
Bark layer × Season	0.186	0.540	0.350
Storage time × Bark layer × Season	0.452	0.225	0.257

**Table 4:** Results (*p*-values) from testing the statistical differences between storage time (i.e., weeks after the treatment onset), season (i.e., winter and summer 2017), and the layers within bark (i.e., inner and outer bark) in antioxidative activities of bark extracts (70% EtOH) of Norway spruce pulpwood logs analyzed with ORAC, FRAP, and SCAV tests. Interaction terms are indicated by x.

	*p*-Values, Pulpwood		
Factor	ORAC	FRAP	SCAV
Storage time	0.000	0.000	0.018
Bark layer	0.323	0.225	0.006
Season	0.014	0.001	0.399
Storage time × Bark layer	0.644	0.357	0.412
Storage time × Season	0.001	0.546	0.052
Bark layer × Season	0.765	0.577	0.043
Storage time × Bark layer × Season	0.421	0.452	0.385

**Table 5:** Tree ages and dimensions of saw log samples of the storage studies. Samples from two stems were taken each sampling time.

	Storage Sample	Sampling Date	Storage Time	Tree Age	Tree Height	D1.3 m		Log Length	Log Diameter					
									Butt End		Middle		Top	
	Number		Weeks	Years	dm	mm	mm	dm	mm	mm	mm	mm	mm	mm
**Winter Storage**	3	7.2.2017	0	119	225	362	345	47	310	319	290	306	261	270
	33	7.2.2017	0	96	223	321	272	46	273	255	242	249	215	227
	12	21.2.2017	2	95	221	282	291	45	257	275	235	251	221	223
	15	21.2.2017	2	110	222	323	345	46	302	291	293	277	255	243
	1	7.3.2017	4	97	210	301	299	47	260	258	234	238	216	219
	2	7.3.2017	4	94	215	280	277	46	282	279	257	263	235	232
	30	2.5.2017	12	73	206	260	263	46	234	226	204	207	191	185
	34	2.5.2017	12	56	213	362	362	45	317	305	282	290	254	262
	14	25.7.2017	24	96	224	323	341	46	264	269	238	244	207	210
	31	25.7.2017	24	78	256	305	299	46	282	281	256	261	247	240
**Summer Storage**	41	30.5.2017	0	67	243	307	310	48	279	272	262	254	243	237
	51	30.5.2017	0	70	245	362	357	44	310	311	300	291	292	289
	47	12.6.2017	2	84	259	360	345	44	300	292	287	285	273	265
	50	12.6.2017	2	108	233	317	327	47	272	287	251	249	232	219
	44	26.6.2017	4	100	270	392	399	43	350	342	324	326	313	305
	49	26.6.2017	4	95	255	360	364	49	303	310	281	284	254	256
	45	22.8.2017	12	94	260	326	326	47	284	290	274	267	290	246
	46	22.8.2017	12	58	225	304	300	48	256	239	225	219	206	199
	42	13.11.2017	24	89	262	359	358	46	329	318	316	305	290	284
	43	13.11.2017	24	93	252	285	278	48	271	260	246	242	226	224

**Table 6:** Tree ages and dimensions of pulpwood samples of the storage studies. Samples from two stems were taken each sampling time.

	Storage Sample	Sampling Date	Storage Time	Tree Age	Tree Height	D1.3 m		Log Length	Log Diameter					
									Butt End		Middle		Top	
	Number		Weeks	Years	dm	mm	mm	dm	mm	mm	mm	mm	mm	mm
**Winter Storage**	22	7.2.2017	0	69	114	121	124	46	133	143	113	113	98	97
	23	7.2.2017	0	65	124	127	131	50	136	146	119	125	102	103
	8	21.2.2017	2	103	114	132	125	48	152	145	122	120	97	97
	29	21.2.2017	2	38	129	151	145	50	153	160	145	136	110	110
	10	7.3.2017	4	86	142	155	153	50	177	175	149	152	130	125
	16	7.3.2017	4	82	125	126	129	51	150	150	120	122	103	103
	18	2.5.2017	12	81	121	129	121	51	180	157	120	115	100	109
	25	2.5.2017	12	50 *	130	130	137	53	153	155	130	129	104	108
	4	25.7.2017	24	87	143	139	140	48	150	153	133	131	118	118
	27	25.7.2017	24	49	114	136	137	51	143	144	126	129	105	101
**Summer Storage**	54	30.5.2017	0	90	117	147	134	51	165	195	134	137	106	109
	56	30.5.2017	0	87	112	119	131	52	141	145	121	120	93	92
	58	12.6.2017	2	62	121	125	119	51	143	140	113	108	90	91
	59	12.6.2017	2	55	100	124	124	52	144	143	112	109	75	79
	55	26.6.2017	4	85	130	157	155	55	184	184	154	143	126	121
	61	26.6.2017	4	70	128	145	137	53	132	136	119	120	98	96
	53	22.8.2017	12	70	127	164	162	51	187	199	159	157	135	136
	60	22.8.2017	12	56	127	147	144	47	186	176	139	143	124	124
	52	13.11.2017	24	79	134	158	170	51	202	193	157	150	132	135
	57	13.11.2017	24	98	153	145	144	51	165	163	140	130	124	115

* Possible inaccuracies in tree age determination due to the sample condition.

## Supplementary Materials

The following are available online: Figure S1: Oxygen radical absorbance capacity (ORAC; a), ferric ion reducing antioxidant power (FRAP; b), and hydrogen peroxide (H_2_O_2_) scavenging activity (SCAV; c) in inner (green) and outer (blue) bark of Norway spruce pulpwood logs after 70% ethanol (70% EtOH) extraction during storage treatments in 2017. Winter and summer samples are pooled together.
